# Development and Evaluation of an Educational E-Tool to Help Patients With Non-Hodgkin's Lymphoma Manage Their Personal Care Pathway

**DOI:** 10.2196/resprot.3407

**Published:** 2015-01-09

**Authors:** Jozette JC Stienen, Petronella B Ottevanger, Lianne Wennekes, Helena M Dekker, Richard WM van der Maazen, Caroline MPW Mandigers, Johan HJM van Krieken, Nicole MA Blijlevens, Rosella PMG Hermens

**Affiliations:** ^1^Radboud university medical centerScientific Institute for Quality of Healthcare (IQ healthcare)NijmegenNetherlands; ^2^Radboud university medical centerDepartment of Medical OncologyNijmegenNetherlands; ^3^Radboud university medical centerDepartment of RadiologyNijmegenNetherlands; ^4^Radboud university medical centerDepartment of RadiotherapyNijmegenNetherlands; ^5^Canisius Wilhelmina HospitalDepartment of Internal MedicineNijmegenNetherlands; ^6^Radboud university medical centerDepartment of PathologyNijmegenNetherlands; ^7^Radboud university medical centerDepartment of HematologyNijmegenNetherlands

**Keywords:** eHealth, personalized care, non-Hodgkin’s lymphoma, patient education, care pathway, consumer health information, empowerment, personal care management

## Abstract

**Background:**

An overload of health-related information is available for patients on numerous websites, guidelines, and information leaflets. However, the increasing need for personalized health-related information is currently unmet.

**Objective:**

This study evaluates an educational e-tool for patients with non-Hodgkin’s lymphoma (NHL) designed to meet patient needs with respect to personalized and complete health-related information provision. The e-tool aims to help NHL patients manage and understand their personal care pathway, by providing them with insight into their own care pathway, the possibility to keep a diary, and structured health-related information.

**Methods:**

Together with a multidisciplinary NHL expert panel, we developed an e-tool consisting of two sections: (1) a personal section for patients’ own care pathway and their experiences, and (2) an informative section including information on NHL. We developed an ideal NHL care pathway based on the available (inter)national guidelines. The ideal care pathway, including date of first consultation, diagnosis, and therapy start, was used to set up the personal care pathway. The informative section was developed in collaboration with the patient association, Hematon. Regarding participants, 14 patients and 6 laymen were asked to evaluate the e-tool. The 24-item questionnaire used discussed issues concerning layout (6 questions), user convenience (3 questions), menu clarity (3 questions), information clarity (5 questions), and general impression (7 questions). In addition, the panel members were asked to give their feedback by email.

**Results:**

A comprehensive overview of diagnostics, treatments, and aftercare can be established by patients completing the questions from the personal section. The informative section consisted of NHL information regarding NHL in general, diagnostics, therapy, aftercare, and waiting times. Regarding participants, 6 patients and 6 laymen completed the questionnaire. Overall, the feedback was positive, with at least 75% satisfaction on each feedback item. Important strengths mentioned were the use of a low health-literacy level, the opportunity to document the personal care pathway and experiences, and the clear overview of the information provided. The added value of the e-tool in general was pointed out as very useful for preparing the consultation with one’s doctor and for providing all information on one website, including the opportunity for a personalized care pathway and diary. The majority of the revisions concerned wording and clarity. In addition, more explicit information on immunotherapy, experimental therapy, and psychosocial support was added.

**Conclusions:**

We have developed a personal care management e-tool for NHL patients. This tool contains a unique way to help patients manage their personal care pathway and give them insight into their NHL by providing health-related information and a personal diary. This evaluation showed that our e-tool meets patients’ needs concerning personalized health-related information, which might serve as a good example for other oncologic diseases. Future research should focus on the possible impact of the e-tool on doctor-patient communication during consultations.

## Introduction

### Overview

In the current digital era, patients are overloaded with health-related information. Many patient associations, health care institutes, hospitals, scientific societies, and guideline working groups provide their own information through websites and flyers. Unfortunately, the increasing call for personalized health-related information is still unmet [1-3]. On the one hand, this information need includes tools that provide support during interaction with caregivers, such as question sheets, decision aids, and option grids [4-6]. These tools aim at providing information about available (treatment) options and possible risks to make a well-informed decision. Decision aids, for example, are shown to be effective with regard to improvement of patients’ involvement and health-related knowledge [7].

On the other hand, patients ask for more insight into their personal care pathway, including diagnostics, therapy, and aftercare [8], which makes it easier to act as managers of their own care. This also points to personalized care, which can be defined as the delivery of care that is tailored to an individual patient. Important elements are (1) the delivery of care that is responsive to individual preferences, needs, values, and possibilities, and (2) as much as possible, patients’ engagement in their own care and health. The latter point needs a well-informed patient, who has insight into his personal care pathway. In the literature, roadmaps or care pathways concerning patient care are mostly directed to professionals [9,10]. However, making these available for patients could help them in their personal care management.

In addition, reliable health-related information is particularly important for patients, where the Internet is an information source of growing importance. A national survey in the United States showed that 59% of adults searched online for health-related information in 2012 [11]. In European countries, over 80% of adults used the Internet as the main source for health-related information in 2011 and 2012 [12,13]. Several quality criteria have been developed worldwide to monitor the quality of easily accessible health-related information on the Internet [14]. The best-known quality criteria are found in the Health On the Net Code of Conduct (HONcode) for websites [15]. Previous research showed that it remains difficult to accurately monitor all information posted on the Internet, for example, online information concerning cancer and other disease-related topics is still of poor quality [16-21].

For non-Hodgkin’s lymphoma (NHL), a heterogeneous group of over 40 types of malignant lymphomas, an abundance of health-related information is available online. Previous research showed that patients diagnosed with NHL would like to have access to more complete and personalized information on diagnostics, therapy, and aftercare [2]. In response to this, we developed a unique e-tool for NHL patients. This study is, to our knowledge, the first description on the development and evaluation of a personalized care pathway for NHL patients that is also linked to the available health-related information concerning NHL.

### Aim and Objectives

This paper describes the development and evaluation of a unique, educational e-tool for NHL patients, aiming to help patients manage and understand their personal NHL care pathway. This is done by providing insight into their personal care pathway based on the data patients enter and by providing essential information about NHL care. Additionally, patients are given the possibility to register personal experiences in their care pathway, as they would in a diary. We hypothesize that having access to all the information available in the e-tool, patients will have a better understanding of their disease and will be able to act as managers of their own care pathways during interaction with their caregivers.

##  Methods

### Setting

The e-tool described in this paper was developed in the context of the PEARL study (improvement of patients’ hospital care for non-Hodgkin’s lymphoma), aimed at improving hospital care for patients diagnosed with NHL [22]. In a previous study, insight into current NHL care was acquired by measuring quality indicators at the patient level [23]. Together with the barriers and facilitators found, as perceived by patients and physicians [2], a tailored improvement strategy was developed. Next to several physician-directed tools, an e-tool for patients was included in the improvement strategy. This paper describes the development and evaluation of an e-tool for NHL patients.

### E-Tool Development

#### Overview

We started with the development of a mock-up storyboard, and with the help of a system developer this was converted into a distinctive e-tool for NHL patients, tailored to address the patients’ barriers found in previous research [2]. The barriers included lack of insight into the patients’ personal care pathway, and lack of written information about diagnostics and therapy. This is why the e-tool developed consists of two sections: (1) a personal section for patients’ own NHL care pathway (roadmap) and their experiences with NHL care, and (2) an informative section including information on NHL. The complete e-tool aims to help patients manage the care they receive during their NHL care trajectory. The webmaster of the Dutch Lymphoma Patient Association (Hematon, known as LVN before 2014) and an expert panel, including a hematologist, radiologist, pathologist, radiation oncologist, epidemiologist, and a senior researcher, were closely involved in the e-tool development. Quality criteria from the HONcode were taken into account during the development process.

#### Personal Section of the E-Tool

In this section, users were able to document dates and experiences of first consultation, diagnostics, and therapy. The aim was to provide insight into the patients’ personal care pathway. We developed a general (ideal) NHL care pathway based on the national guidelines and recommendations available in the Netherlands. These included, among others, the Dutch NHL guidelines, recommendations of the Cooperative Trial Group for Hematology Oncology (HOVON) and the Dutch Society of Hematology (NVvH), general recommendations for acceptable waiting times, and an NHL guideline based on patients’ perspectives of Hematon. Internationally available guidelines were consulted when applicable. The ideal care pathway, including date of first consultation, date of diagnosis, and start date of therapy, was used to set up the format for the personal care pathway (roadmap).

#### Informative Section of the E-Tool

Users had access to reliable information on NHL and NHL care through this section of the e-tool. The aim was to cluster all reliable, online available information and make it understandable for all users. Too much or confusing information and a high health-literacy level were avoided as much as possible. The Dutch NHL guidelines, NVvH, HOVON, the Dutch Cancer Society (KWF), and several NHL-related websites (eg, Radiotherapie Nederland, Chemo and nu, Hematon, and Hematologie Groningen) were used as sources of NHL information for the content. These sources are frequently recommended and used by professionals, so the content can be considered as authorized by them. We cooperated with the webmaster of Hematon to make sure that the information included was complete and accurate. The format of the e-tool was based on the NHL care pathway as described in the guidelines and on user experiences with other websites.

### E-Tool Evaluation

After evaluation of the concept of the text by the expert panel, an external hematologist, and the webmaster of Hematon, an assessment was set up to evaluate both sections of our e-tool concerning applicability. We recruited NHL patients via Hematon and a university hospital in the Netherlands. Patients were eligible when they had, or have had, a non-Hodgkin’s lymphoma, were over 18 years old, and had access to the Internet. Family and friends of 3 members of the project team were asked to participate as laymen. The e-tool was evaluated by patients and laymen using a 24-item questionnaire—19 yes/no questions, including space for reasoning, and 5 open-ended questions, including improvement suggestions. The questionnaire included questions on layout (6 questions), user convenience (3 questions), menu clarity (3 questions), information clarity (5 questions), and general impression (7 questions), for example, strengths and missing information. This method allowed us to obtain as much information as possible to improve our e-tool. The expert panel was invited by email to give general feedback on the e-tool.

## Results

### E-Tool Development

#### Overview

We developed an e-tool, consisting of two sections—a personal section and an informative section. The content of the sections will be described below, followed by the results of the evaluation of the e-tool applicability. The e-tool is available in Dutch [24] for participants of the PEARL study and will become publicly available in 2015. The e-tool started with a general introduction, which described the aim of the e-tool and gave a short overview of the personal and informative sections of the e-tool. Both sections were formatted in chronological order (diagnostics, therapy, aftercare), which is in line with the NHL care pathway as described in the guidelines and seen in clinical practice. Additionally, background information was available concerning the PEARL study, participating hospitals, and contact information.

#### Personal Section of the E-Tool

This section consisted of questions concerning a patient’s personal NHL care pathway, which allowed patients to develop a unique overview of all diagnostics, treatments, and aftercare/follow-up that they have had. Questions were divided into four items:

1. Diagnostic examinations, including items such as type and date of the examination, executed by whom, and in which hospital.

2. Diagnosis, including date of first consultation, date of diagnosis, type of NHL, stage of the disease, risk profile, date of treatment plan, hospital name, and physician name.

3. Therapy, including type and date of therapy, executed by whom, and in which hospital.

4. Aftercare, including items such as type and date of aftercare, executed by whom, and contact information.

**Figure 1:**
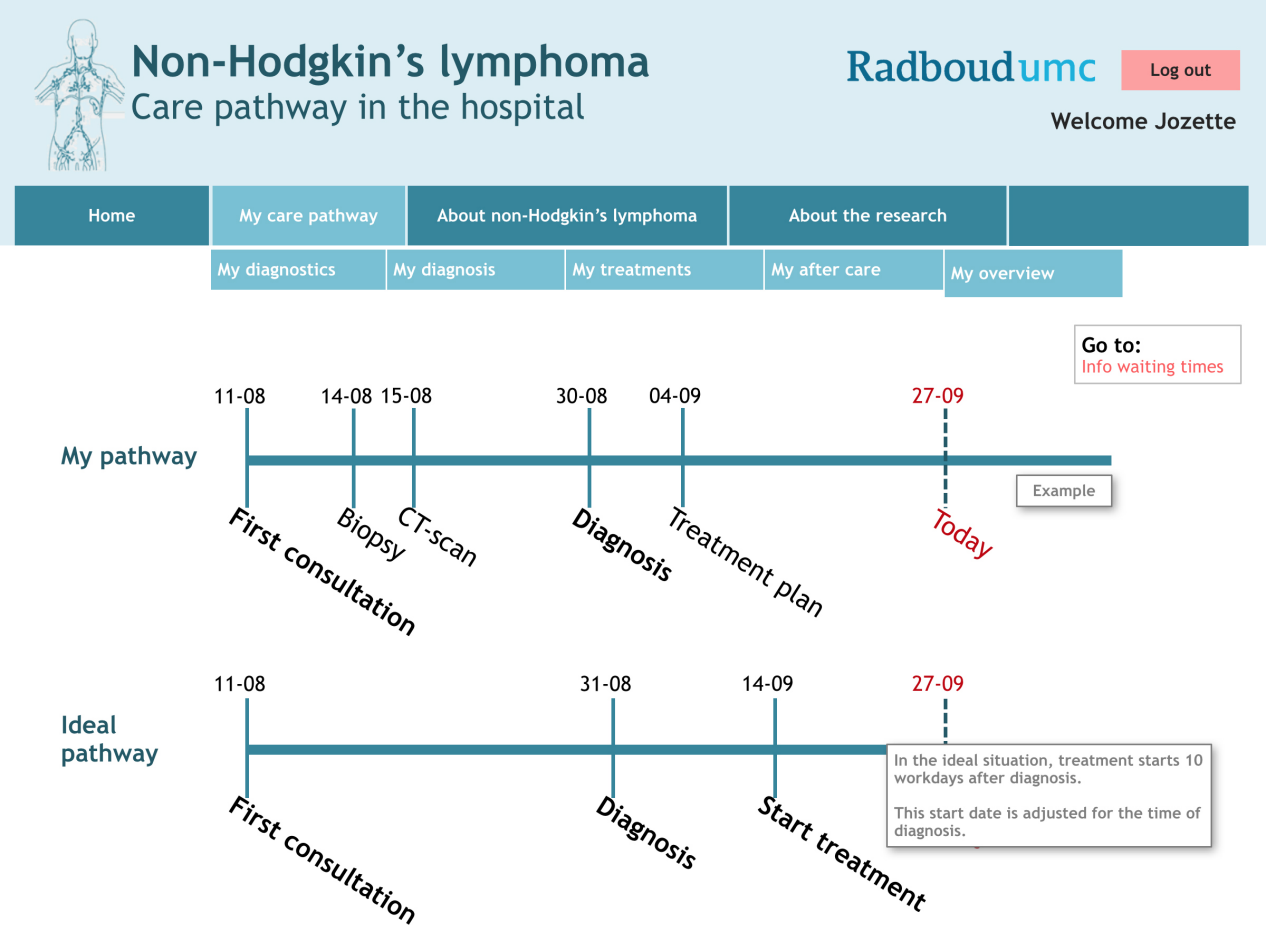
Example of a personal NHL care pathway (personal section of the e-tool). Original is in Dutch.

An easy link from the personal section to the informative section was provided next to each item. In this way, patients could look up the underlying information belonging to the different items. Additionally, it was possible to create a diary, to document their own experiences with the different diagnostic examinations or treatments. Information filled out in this diary was only visible to the patients themselves. Finally, an overview of the personal NHL care pathway was obtained using the answers to the questions. The personal diary—chronologically visualized experiences with NHL care—was provided when applicable. An example of the personal NHL care pathway (roadmap) is shown in Figure 1.

#### Informative Section of the E-Tool

This section consisted of NHL information from reliable online sources, divided into six information items:

1. NHL in general, including a description of what NHL is, symptoms, and most occurring NHLs.

2. Diagnostic examinations, including items such as blood sample, tissue biopsy, bone marrow biopsy/aspirate, PET/CT-scan, and additional examinations.

3. Diagnosis, including stage, international prognostic index (IPI) score, treatment plan, and additional support.

4. Therapy, including wait-and-see, radiotherapy, chemotherapy, immune therapy, stem cell transplantation, and clinical trials.

5. Aftercare, including response evaluation and monitoring.

6. Waiting times.

**Figure 2:**
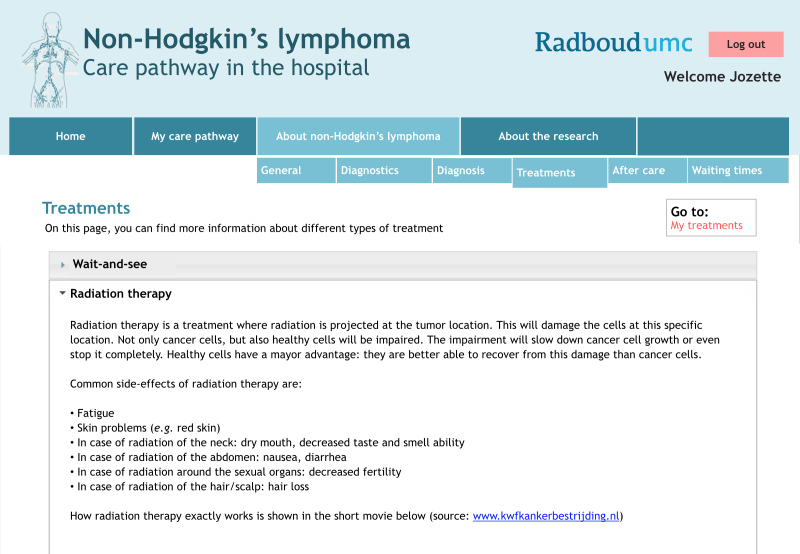
Example of informative section of the e-tool. Original is in Dutch.

External links for more extensive information were added as much as possible, together with short educational movies created by KWF or Hematon. An example of the informative section is shown in Figure 2.

### E-Tool Evaluation

We asked 20 people to participate in our test, including 14 NHL patients and 6 laymen. In total, 13 people logged in and 12 returned the questionnaire, which resulted in a 60% (12/20) response rate. Little feedback was given by 1 participant by email and he did not return the questionnaire. The mean age of the 12 participants (patients and laymen) that completed the questionnaire was 56 years (SD 11), with a range of 30-70 years. Of the participants, 42% (5/12) were female and 67% (8/12) had a high education level (college or university). Additionally, all 6 panel members and the external hematologist provided general feedback by email.

Table 1 includes the results of our e-tool evaluation (applicability) using closed questions among NHL patients and laymen. Overall, the evaluation of the e-tool was highly positive. Table 1 shows at least 75% (9/12) satisfaction on each section. Concerning layout, all responders were satisfied with the clarity of the text, writing style, and clarity of the illustrations and short videos. When looking at the user convenience, 11 out of 12 participants (92%) were satisfied with the format and speed of the e-tool. In relation to menu clarity, all participants found the menu format intuitive and the information needed was easy to find. Information clarity, including understandability of the text, was evaluated positively as well. Only 2 out of 12 participants (17%) thought too much medical terminology was included.

**Table 1:** Results of the e-tool evaluation among NHL patients and laymen (closed questions).

Question		Positive response, n (%)	
		Patients (n=6)	Laymen (n=6)
**Layout**			
	Is the text clear?	6 (100)	6 (100)
	Are you satisfied with letter type and size?	3 (50)	6 (100)
	Are you satisfied with the writing style?	6 (100)	6 (100)
	Is the use of colors attractive to you?	4 (67)	5 (83)
	Are there enough illustrations and short videos to support the text?	5 (83)	5 (83)
	Are the illustrations and short videos clear?	6 (100)	6 (100)
**User convenience**			
	Is the e-tool easy to use?	4 (67)	5 (83)
	Is the format of the e-tool easy to understand?	5 (83)	6 (100)
	Are you satisfied with the speed of the e-tool?	6 (100)	6 (100)
**Menu clarity**			
	Does the composition of the menu seem natural?	6 (100)	6 (100)
	Are you satisfied with the navigation through the e-tool?	5 (83)	5 (83)
	Is it easy to find the information you are looking for?	6 (100)	6 (100)
**Information clarity**			
	Is the information provided on the e-tool understandable?	6 (100)	6 (100)
	Does the e-tool make use of too much medical terminology?^a^	4 (67)	6 (100)
	Do you understand what NHL is, after reading the information provided on the e-tool?	6 (100)	6 (100)
	Do you understand what treatment options are available, after reading the e-tool?	6 (100)	6 (100)
**General impression**			
	Are there any flaws/errors in this e-tool?^a^	5 (83)	5 (83)
	Would you use this e-tool if made available to you?	5 (83)	6 (100)
	Are there any redundant items in this e-tool?^a^	6 (100)	6 (100)

^a^Percentage of negative responses presented, caused by negative questioning.

Table 2 includes the results of our e-tool evaluation using open-ended questions, and shows mainly positive remarks about the e-tool and some improvement points, according to NHL patients and laymen. Valuable strengths mentioned by the participants included the use of a low health-literacy level, the opportunity to document their personal care pathways and experiences (diary), and the clear and helpful overview of the information provided. The added value of the e-tool in general was pointed out as very useful for preparing for their consultations with their doctors and for providing all information in one website, including the opportunity for a personalized care pathway and diary.

In general, the e-tool is considered to be a good initiative to help patients manage their NHL care. For example, one participant wrote, “I think this tool is excellent and that it will be helpful in dealing with the fact that you are diagnosed with NHL. The tool can contribute to an optimal doctor-patient contact.” There were no distinct differences in feedback between patients and laymen, except that patients provided more comments concerning missing items and general remarks.

Improvement points proposed by the expert panel included addition of a search function and contact button, adding items to the personal care pathway section, suggestions for links to general cancer websites, and some wording issues (data not shown).

During the review of the e-tool, all feedback was taken into account. The majority of the revisions concerned wording and clarity. With respect to layout, the font size regarding explanatory text was enlarged and the color of the submenu was made less bright. Regarding user convenience and menu clarity, the navigation at the bottom of each page was made clearer. No revisions were made concerning information clarity. In addition, more explicit information was included about immune therapy, experimental therapy, and psychosocial support. As well, items for the patients’ risk profiles and stages of disease were added to the personal section.

**Table 2:** Results of the e-tool evaluation among NHL patients and laymen (open-ended questions).

Question		Response
**Information clarity**		
	What does this e-tool add to the information provided by your doctor?	Makes everything much clearer and understandable. Makes it possible to read everything at a quiet moment. Provides good preparation for consultation with your doctor. Provides a great overview of all necessary information. Provides a good overview of your own care pathway. The e-tool is very useful for the patients’ relatives. Makes it possible to compare with the ideal pathway.
**General impression**		
	What are improvement points for this e-tool?	More expressive color use. For patients that forget updating their care pathway, are there alerts? Better navigation through the care pathway. Clearer definition of first consult.
	What are the strengths of this e-tool?	Opportunity to document your personal care pathway. Opportunity to document your experiences in a diary. Possible to compare with quality criteria (ideal pathway). Low health-literacy level. Clear and calm colors. Clear overview of medical information and terminology. Clear layout and navigation. External links to additional information about NHL. Overview of information gives the opportunity to translate this to yourself.
	What did you miss on this e-tool?	Overview of lymph vessels. Information about immune therapy and clinical trials. Information about psychosocial support. Information about second opinion.
	General remarks?	Great overview of available NHL information. Other websites are fine, but this e-tool provides more specific information. I already have my own diary, which I will use in the future. The tool gives the opportunity to create your own care pathway and diary—this has a positive influence on dealing with the fact that you are diagnosed with NHL. This e-tool can contribute to an optimal doctor-patient contact during consultations.

## Discussion

### Principal Results

In this study, we showed the development and evaluation of a unique e-tool for, and by, patients with non-Hodgkin’s lymphoma. Based on the evaluation, the e-tool is a feasible tool that gives patients insight into their personal care pathway and informs them about NHL. The overall positive feedback implies the e-tool’s added value of providing personalized health information, which is often an unmet need for patients. Taken as a whole, this is the first e-tool in the field of oncology that aims at helping patients in their personal care management and provides an additional overview of information about NHL.

### Comparison With Prior Work

Involving patients in the development of health care improvement tools has been an upcoming phenomenon in the past several years. Nowadays, the patient’s voice is incorporated more often in clinical guidelines, quality indicators, and shared decision-making instruments [8,25,26]. It is important to support patients involved in developing health care improvement tools. For example, a wiki has been established for, and tested by, patients involved in the development of a guideline on infertility [27]. The shared decision-making instruments themselves also have a supporting role, as they try to help patients in making informed decisions [6,28-30]. However, not all decision aids are directed to patients [31,32] and they usually do not provide insight into the complete personal care pathway.

Studies focusing on personalized care pathways and assistance with patients’ personal care management are sparsely described in the literature. Most studies concerning the development of patient care pathways or roadmaps concentrate on the education of physicians [33,34] or other health care professionals [10]. Ryhänen et al [35] and Dykes et al [36] developed tools to educate patients about their care pathway. Dykes et al aimed at providing education about the anticipated length of stay and treatment plan after acute myocardial infarction. Development and evaluation of a breast cancer pathway, an Internet-based patient education program, was described by Ryhänen et al. In line with our e-tool, these studies focused on patient education concerning the patient care pathway. However, these studies did not include information tailored to the individual patient, such as a chronological overview of the personal care pathway or a diary. Atack et al [37] developed an online patient education system including health-related information tailored to the individual patient. Physicians were able to “prescribe” information that met the patient’s need. However, this system was not focused on the total care pathway and was only pilot-tested in 8 patients. Our e-tool is the first that combines the unmet needs of patients, including personalized information about the patients’ care pathways and the possibility to create a diary, together with an overview of all necessary NHL information.

### Limitations

Four limitations were identified with respect to this e-tool. First, this e-tool has been developed for NHL patients, a patient category with a more advanced age—incidence of NHL increases with age—that might not be active on the Internet. However, the mean age of NHL patients included in this study was 61 years (SD 7), and a previous study on possible barriers in NHL care also showed inclusion of patients of all ages [2]. Furthermore, health-related Internet use seems to increase over time in Europe among people of all ages [38]. Second, the questionnaire used to evaluate the e-tool was not validated, but based on evaluation factors derived from the human, organization, and technology-fit (HOT-fit) framework [39]. We believe that the questionnaire developed included all necessary items to extensively evaluate the e-tool.

Third, the evaluation study only assessed feasibility of the e-tool on a small scale. Unfortunately, no information was available about the 7 nonresponders, and a relatively high percentage of the responders were highly educated, which could have introduced some bias. A next step in the implementation is to test the e-tool in daily NHL care and evaluate user experiences on a larger scale. This might give insight into the possibilities for making the e-tool appropriate on an international level and it might serve as a good example for other oncologic diseases.

Finally, the e-tool is not yet certified by the HONcode. However, all eight items of the code of conduct for medical and health websites as described by HON were taken into account during the development of the e-tool [40]. After testing the e-tool at a larger scale we intend to apply for certification.

### Conclusions

We have developed a personalized approach using an e-tool for NHL patients. This tool contains a unique way to help patients with their care management—it provides insight into the personal care pathway and offers general information about NHL. In this evaluation study, we report high satisfaction rates and some improvement points for future use. We expect that the e-tool will have a positive impact on both patient empowerment and doctor-patient communication, since patients are more informed in lay language about NHL and their personal care pathway. It is suggested that better-informed patients are able to ask more specific questions, which makes it possible to improve the management of their personal care. Finally, this e-tool may play an important role in dealing with NHL, particularly the personal diary option, which has the potential to provide psychosocial support for personal experiences in NHL care. The usage and effects of the e-tool should be tested and evaluated in future research, which is included as one of the purposes of the PEARL study [22].

## Abbreviations

HONcode: Health On the Net Code of Conduct
HOT-fit: human, organization, and technology-fit
HOVON: Cooperative Trial Group for Hematology Oncology
IPI: international prognostic index
KWF: Dutch Cancer Society
NHL: non-Hodgkin’s lymphoma
NVvH: Dutch Society of Hematology
NWO: Dutch Organization for Scientific Research
PEARL: improvement of patients’ hospital care for non-Hodgkin’s lymphoma
ZonMW: Netherlands Organization for Health Research and Development
